# Population-scale predictions of DPD and TPMT phenotypes using a quantitative pharmacogene-specific ensemble classifier

**DOI:** 10.1038/s41416-020-01084-0

**Published:** 2020-09-25

**Authors:** Yitian Zhou, Carolina Dagli Hernandez, Volker M. Lauschke

**Affiliations:** 1 Department of Physiology and Pharmacology, Karolinska Institutet, 17177 Stockholm, Sweden; 2 Department of Clinical and Toxicological Analyses, School of Pharmaceutical Sciences, University of Sao Paulo, 05508-000 Sao Paulo, Brazil

**Keywords:** Cancer genetics, Cancer genomics, Cancer genetics

## Abstract

**Background:**

Inter-individual differences in dihydropyrimidine dehydrogenase (*DPYD* encoding DPD) and thiopurine S-methyltransferase (TPMT) activity are important predictors for fluoropyrimidine and thiopurine toxicity. While several variants in these genes are known to decrease enzyme activities, many additional genetic variations with unclear functional consequences have been identified, complicating informed clinical decision-making in the respective carriers.

**Methods:**

We used a novel pharmacogenetically trained ensemble classifier to analyse *DPYD* and *TPMT* genetic variability based on sequencing data from 138,842 individuals across eight populations.

**Results:**

The algorithm accurately predicted in vivo consequences of *DPYD* and *TPMT* variants (accuracy 91.4% compared to 95.3% in vitro). Further analysis showed high genetic complexity of DPD deficiency, advocating for sequencing-based *DPYD* profiling, whereas genotyping of four variants in *TPMT* was sufficient to explain >95% of phenotypic TPMT variability. Lastly, we provided population-scale profiles of ethnogeographic variability in DPD and TPMT phenotypes, and revealed striking interethnic differences in frequency and genetic constitution of DPD and TPMT deficiency.

**Conclusion:**

These results provide the most comprehensive data set of *DPYD* and *TPMT* variability published to date with important implications for population-adjusted genetic profiling strategies of fluoropyrimidine and thiopurine risk factors and precision public health.

## Background

Adverse drug reactions (ADRs) are a common phenomenon in cancer therapy, and the identification of patients at increased risk thus constitutes an important goal of precision oncology. In the last decade, genetic profiling has identified a multitude of variations that can guide selection and dosing of chemotherapeutic drugs.^1^ Two of the most important examples of such pharmacogenetic biomarkers that have transitioned from research into clinical practice are germline variations in the dihydropyrimidine dehydrogenase (*DPYD* encoding DPD) and thiopurine S-methyltransferase (*TPMT*) genes.^2–4^

Fluoropyrimidines are cornerstones of oncological therapy used for the treatment of a wide range of solid tumours. Importantly, DPD deficiency is strongly associated with dose-limiting and sometimes life-threatening toxicity with 60–80% of DPD-deficient individuals experiencing severe ADRs compared to 10–20% of patients with normal enzyme function.^5,6^ The most extensively studied variation associated with DPD deficiency is DPYD*2A (rs3918290), a splice donor variant that results in truncated protein without catalytic activity.^7^ Recent meta-analyses moreover confirmed robust associations of *DPYD* I560S, D949V as well as of the intronic splice variant rs75017182 and the associated haplotype HapB3 with fluoropyrimidine toxicity,^8–10^ and prospective testing for these variants followed by genotype-guided upfront dose adjustments significantly increased patient safety.^11–13^ Analogously to *DPYD*, individuals deficient in TPMT are more susceptible to life-threatening toxicity of thiopurines.^14^ The most important decreased function alleles are TPMT*2 (rs1800462), *3A (rs1800460 and rs1142345) and *3C (rs1142345).^15^

In addition to the well-characterised variants illustrated above, *DPYD* and *TPMT* harbour hundreds of additional rare genetic variations with unclear effects on enzyme function.^16,17^ Recent advances in large-scale mutagenesis screens unlock exciting opportunities for the parallel experimental interrogation of the effect of thousands of variants,^18^ as exemplified by the simultaneous characterisation of the effects of thousands of *TPMT* variants on intracellular abundance.^19^ However, without experimental assessments of variant effects on enzyme activity, their interpretation has to rely on computational tools. In the last two decades, a multitude of computational prediction tools have been developed that consider sequence conservation as an indicator of variant deleteriousness, as well as various mechanistic parameters, such as impacts on physiochemical properties, post-translational modifications and structural features, such as protein stability and the disruption of binding interfaces.^20,21^ These algorithms are mostly trained on pathogenic variants for which evolutionary conservation constitutes a suitable proxy.^22^ However, evolutionary constraints for *DPYD* and *TPMT* are limited, and conservation scores are thus not the ideal metric to predict variant function. To overcome these problems, we recently established a computational framework optimised specifically for pharmacogenomic predictions.^23^

Using this algorithm, we here mapped *DPYD* and *TPMT* variants on an unprecedented scale using whole-genome sequencing (WGS) and whole-exome sequencing (WES) data from 138,842 individuals. Using all variations with known in vivo consequences as benchmark data set, we show that our pharmacogenetically trained ensemble classifier substantially outperforms all previous non-gene-specific prediction methods and achieved predictive accuracy similar to in vitro experiments (accuracy 91.4% compared to 95.3% for in vitro). Encouraged by these results, we applied the algorithm to the functional interpretation of all 859 identified *DPYD* and *TPMT* variants that affect the amino acid sequence of their respective gene products. Our results reveal that interrogation of only four variants is sufficient to identify >95% of *TPMT*-decreased function alleles, whereas 174 variations have to be profiled for *DPYD* to explain the same level of genetically encoded functional variability. Furthermore, we demonstrate substantial differences in metabolic activity and the underlying genetic variability across eight different populations with important implications for the design of pharmacogenetic testing strategies and precision public health.

## Methods

### Data collections and definitions

In vitro data of missense single-nucleotide variants (SNVs) in *DPYD* and *TPMT* were collected from functional studies conducted using cell lines.^7,24–33^ As classification criteria for variants differed between studies, we homogenised the definitions and considered all variations as deleterious, which resulted in in vitro activity lower than 70% of the respective wild-type (WT) enzyme. In vivo data were collected from the ClinVar database.^34^ The deleteriousness of variants was curated based on their annotation as impacting drug response, pathogenic or likely pathogenic. Variant frequencies from 138,842 individuals across eight different populations (12,487 Africans, 17,720 Latinos, 5185 Ashkenazi Jews, 9977 East Asians, 64,603 Non-Finnish Europeans, 12,562 Finnish, 15,308 South Asians and 1000 Swedes) were collected from GnomAD^35^ and SWEgene.^36^ Linkage between the *TPMT* variants rs1800460 and rs1142345 was calculated using LDlink.^37^

### Variant-effect predictions

We used Polyphen-2, SIFT, PROVEAN and CADD for binary predictions of variant functionality. To quantitatively predict the functional impact of all identified variants, we used the ADME-optimised prediction framework (APF) that provides normalised quantitative functionality prediction scores in the range from 1 (neutral) to 0 (deleterious).^23^ The functional impact of frameshift and stop-gain variations was further confirmed by LOFTEE (https://github.com/konradjk/loftee). For qualitative comparisons with binary scores, variants with functionality scores ≤0.5 were considered as deleterious, while scores >0.5 denote functionally neutral variants.

### Informedness calculations

Plotting the cumulative number of variants against their cumulative aggregated frequencies reveals the excess of information that can be obtained for each number of variants tested. The informedness (*I*) of *DPYD* or *TPMT* testing is defined as the maximal vertical difference between this receiver-operating characteristic (ROC) curve and the bisectrix through the origin of the coordinate system. The intronic variant rs75017182 that is linked to haplotype HapB3 was included with a functionality score of 0.5 according to current guidelines.^38^

### Calculation of DPD and TPMT metaboliser phenotype frequencies

To calculate the frequencies of poor metabolisers (PM), intermediate metabolisers (IM) and normal metabolisers (NM) for each population analysed, we calculated all diplotype frequencies for *DPYD* and *TPMT* and added the functionality scores of both alleles to yield the corresponding activity score of the individual. PMs, IMs and NMs were defined as activity scores (AS) of ≤0.5, 0.5 < AS ≤ 1.5 and >1.5, respectively, according to the genotype combinations defined by Clinical Pharmacogenetics Implementation Consortium (CPIC) guidelines.^38,39^

## Results

### Benchmarking of the ADME prediction framework on *DPYD* and *TPMT* variants with known in vivo consequences

**Fig. 1:**
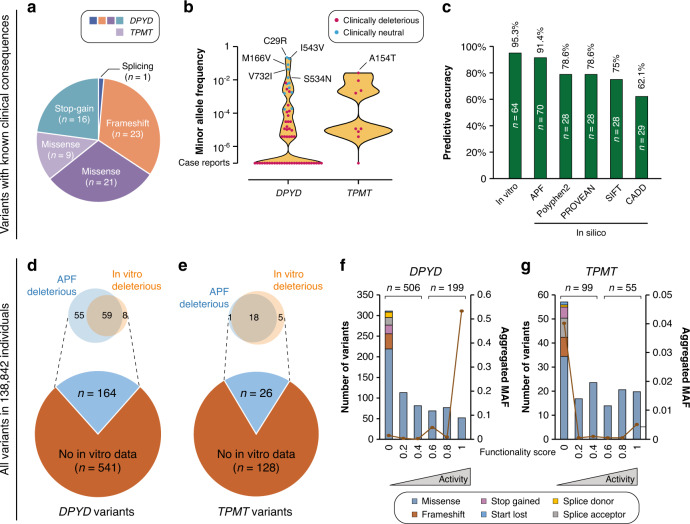
The ADME prediction framework (APF) constitutes an accurate and scalable tool for functional *DPYD* and *TPMT* variant interpretation. **a** Overview of the benchmarking data set, comprising a total of 70 variations (*n* = 61 in *DPYD* and *n* = 9 in *TPMT*) with known clinical consequences. **b** The vast majority of variants resulting in decreased enzyme function (indicated in red) were rare, whereas functionally neutral variants (indicated in blue) were predominantly common with minor allele frequencies (MAF) > 1%. **c** In vitro tests were most accurate, correctly predicting the functional consequences of 95.3% of analysed variants, closely followed by predictions using APF (91.4%). The accuracy of other computational methods was substantially lower (62.1–78.6%). Based on genomic data from 138,842 individuals, 705 and 154 coding variants in *DPYD* (**d**) and *TPMT* (**e**) were identified. Pie charts show that in vitro data were only available for a small fraction of these. Venn diagrams indicate that in vitro tests and APF predictions agree for the majority of those variations. The majority of the number of identified variations was predicted to abolish DPD (**f**) and TPMT (**g**) enzyme function. For *DPYD*, functionally neutral variations were most common, whereas no common neutral variants were identified for *TPMT*. Columns indicate the number of variations, while line plots show the aggregated frequencies of variants with the respective functionality score.

To test the predictive power of the APF algorithm, we first defined a gold-standard data set for *DPYD* and *TPMT* that included all variants with well-characterised functional effects in vivo in humans. Extensive literature search revealed a total of 61 variants with known clinical consequences in *DPYD*, of which 12 were classified as neutral and 49 as pathogenic (Supplementary Table 1). For *TPMT*, only nine variants were sufficiently studied for their impact on drug response in vivo, all of which were deleterious. For *DPYD*, these characterised variants resulted in frameshifts (*n* = 23), followed by missense (*n* = 21), stop-gain (*n* = 16) and splice variants (*n* = 1; DPYD*2A), whereas all nine characterised *TPMT* variants were missense (Fig. 1a). Of all 70 clinically annotated *DPYD* and *TPMT* variants, six were common with global minor allele frequencies (MAF) > 1% (C29R, I543V, M166V, V732I and S543N in *DPYD* and A154T in *TPMT*), whereas all other variants were rare (Fig. 1b).

For variants for which in vitro data were available (64 out of 70 variants, 91%), these experimental categorisations were in good agreement with in vivo phenotypes (95.3% accuracy, 61 out of 64 variants, Fig. 1c). The APF could analyse all 70 variants and achieved 91.4% accuracy (64 out of 70), thus closely approximating the accuracy of in vitro testing. In contrast, other commonly used in silico tools, such as SIFT, Polyphen-2, PROVEAN and CADD, had substantially lower predictive power (62.1–78.6% accuracy) and failed to predict the functional consequences of >50% of variations. Only one incorrectly classified variant overlapped between in vitro and in silico assessments. R215H in *TPMT* (TPMT*8) was incorrectly predicted as benign by APF and in vitro data, while it is associated with reduced TPMT activity in vivo.^40^ Notably, however, as this allele is very rare (MAF = 0.2%), TPMT enzyme activity in vivo has to the best of our knowledge only been reported for a single carrier.

### Population-scale prediction of *DPYD* and *TPMT* variant functionality

As the predictive accuracy of APF exceeded 90% and was similar to in vitro testing on the gold-standard in vivo data set, we expanded our evaluations and tested *DPYD* and *TPMT* variability on a population scale (Fig. 1d–g). By analysing WGS and WES data from 138,842 individuals, we identified 705 and 154 *DPYD* and *TPMT* variants, respectively, of which only 164 (23%) and 26 (17%) had been analysed in cell systems (Fig. 1d, e). Of the 164 experimentally tested *DPYD* variants, in vitro tests predicted that 67 (41%) decreased enzyme function, whereas the fraction was considerably higher using computational tests (*n* = 114, 69.5%, Fig. 1d). In contrast, the number of variants predicted to be deleterious by in vitro assays was substantially higher for *TPMT* where 23 out of 26 tested variants (88%) resulted in functional consequences, while only 19 variants (73%) were predicted to be deleterious by APF (Fig. 1e).

Lastly, we predicted the functional effects of all identified variants, including those without available in vitro data. In total, 506 out of 705 *DPYD* variants (72%) were predicted to be deleterious with functionality scores below 0.5, of which 311 were LOF variants with <20% of WT enzyme activity (Fig. 1f). For *TPMT*, 99 and 55 out of 154 variants (64 and 36%, respectively) were predicted to be deleterious and neutral, respectively (Fig. 1g). The highest frequency of all alleles was for the loss-of-function allele TPMT*3A with a MAF = 2.8%.

### Genetic complexity of DPD and TPMT function

**Fig. 2:**
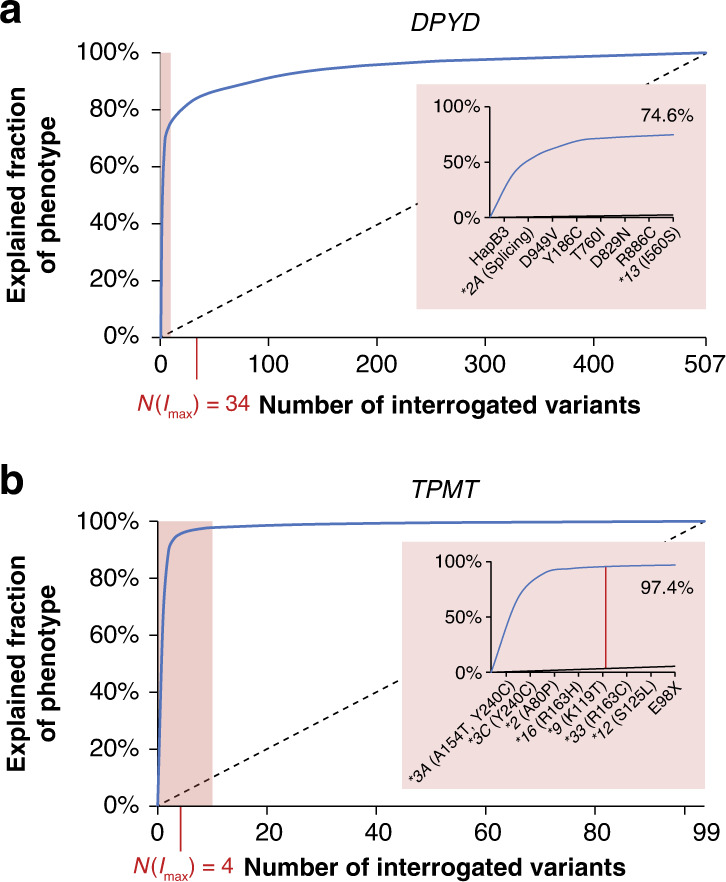
The genetically encoded functional complexity of DPD and TPMT is distinctly different. Receiver-operating characteristic (ROC) curves showing the fraction of functional variability that can be explained by a given number of *DPYD* (**a**) and *TPMT* (**b**) variants. All deleterious variants (functionality score ≤ 0.5) were sorted by the global minor allele frequency. Informedness was determined graphically for every number of variants as the vertical distance between the ROC curve and the bisectrix (dashed line). The number of variants for which the informedness is maximal is indicated as N(*I*_max_) in red. Inlets specify the contribution of the seven variants that explain most of the putative differences in enzyme function. Note that while the eight most frequent variants explain 97.4% of genetically encoded functional variability in TPMT, the same number explains only 74.6% for DPD.

We then evaluated the distribution of *DPYD* and *TPMT* genotypes across reduced function alleles. Specifically, we calculated the fraction of reduced function phenotypes that could be explained by selections of variants in the respective genes. For *DPYD*, 50% of reduced function alleles are explained by two variants (HapB3 and *2A), whereas the numbers of variants that need to be interrogated to explain more of the functional variability increase exponentially (Table 1). For instance, inclusion of six more variants only explains an additional 24.6% of DPD functionality, whereas 88 and 421 variants need to be interrogated to explain 90 and 99% of DPD deficiency, respectively (Fig. 2a and Table 1). The highest excess of information is obtained for the testing of 34 variations, which can explain 84.2% of genetically encoded functional DPD variability while only corresponding to 6.7% of deleterious *DPYD* variants.

**Table 1:** Number of variants needed to explain the functionality of DPD and TPMT.

	Number of variants (% of all deleterious variants) that need to be interrogated	
Functionality explained by variants	*DPYD*	*TPMT*
50%	2 (0.4%)	1 (1%)
70%	5 (1%)	2 (2%)
90%	88 (17.4%)	3 (3%)
95%	174 (34.3%)	4 (4%)
99%	421 (83%)	28 (28.3%)
100%	507 (100%)	99 (100%)
Maximal informedness	34 (6.7%)	4 (4.0%)

*DPYD* encoding DPD dihydropyrimidine dehydrogenase, *TPMT* thiopurine S-methyltransferase.

In comparison to *DPYD*, functional variability in *TPMT* was characterised by a high excess in information allotted to only few variations. Interrogation of A154T and Y240C (TPMT*3), corresponding to 2% of all deleterious *TPMT* variants, was sufficient to explain >70% of functional TPMT functional variability (Fig. 2b and Table 1). Furthermore, >95% of differences in TPMT function were attributed to only four variants (A154T, Y240C, A80P and R163H corresponding to *3A, *3C, *2 and *16), whereas the remaining 95 variants combined only accounted for 4.6%. Notably, while previous studies also reported *3A, *3C and *2 explaining 90–95% of TPMT-deficient phenotypes,^41^ our finding underscored the clinical relevance of TPMT*16, a missense variant with frequencies of up to 0.5% in Scandinavia populations. Overall, these findings reveal that the genetically encoded functional variability in *DPYD* is considerably more complex than for *TPMT*. These findings have important implications for genotype-guided prescribing, as comprehensive sequencing of *DPYD* is necessary to assure the identification of variations impacting fluoropyrimidine toxicity, whereas the genotyping of only a few selected candidate variations is sufficient to explain the vast majority of TPMT variability to inform prescription and dosing of thiopurines.

### Ethnogeographic differences of clinically important *DPYD* and *TPMT* allele frequencies

Reduced function variations of *DPYD* are highly population specific (Table 2). HapB3 variant is overall most frequent and is common in European populations with a MAF of 2.1%. Similarly, the frequency of DPYD*2A is the highest in Northern Europe, particularly in Finland (MAF = 2.4%) and Sweden (MAF = 0.8%), whereas it is very rare (MAF ≤ 0.1%) in Africans, Latinos and East Asians. In contrast, the majority of reduced function alleles in Africans are allotted to the population-specific variants Y186C, A450V and V732G with MAFs of 2.2%, 0.3% and 0.2%, respectively. In South Asians, T760I constitutes the most common variant impacting DPD function (MAF = 0.5%), whereas this variant is absent in all other populations studied, including East Asians.

**Table 2:** Ethnogeographic differences in reduced function variants of DPD and TPMT.

				MAF (%)								
Gene	RSID	Star allele	Variant type	Global	EUR	AFR	FIN	SWE	EAS	SAS	LAT	AJ
*DPYD*												
	rs75017182	HapB3	Intronic	1.3	2.1	0.2	1.2	1.9	0.2	N.A.	0.8	0.7
	rs3918290	*2A	Splicing	0.6	0.6	0.1	2.4	0.8	0	0.4	0.1	0.5
	rs115232898		Missense (Y186C)	0.2	<0.1	2.2	0	0	<0.1	<0.1	<0.1	0
	rs67376798		Missense (D949V)	0.3	0.5	<0.1	<0.1	0.5	<0.1	<0.1	0.3	<0.1
	rs112766203		Missense (T760I)	<0.1	0	0	0	0	0	0.5	0	0
	rs72975710		Missense (A450V)	<0.1	<0.1	0.3	0	0	0	0	<0.1	0
	rs60511679		Missense (V732G)	<0.1	<0.1	0.2	0	0	0	0	<0.1	0
	rs59086055		Missense (R592W)	<0.1	<0.1	<0.1	0	0	0.2	<0.1	0	0
	rs769167857		Splicing	<0.1	0	0	0	0	0	0.1	0	0
	rs189768576		Stop gain (R74X)	<0.1	0	0	0	0	0.2	<0.1	<0.1	0
*TPMT*												
	rs1142345 and rs1800460	*3A	Missense (Y240C, A154T)	2.8	3.8	0.7	2.7	3.8	<0.1	0.7	4.3	1.3
	rs1142345	*3C	Missense (Y240C)	1	0.4	4.8	0.3	0.2	1.4	1.1	0.5	<0.1
	rs1800462	*2	Missense (A80P)	0.2	0.2	<0.1	<0.1	0.2	0	0	0.4	0.2
	rs144041067	*16	Missense (R163H)	<0.1	<0.1	<0.1	0.5	0.1	<0.1	<0.1	<0.1	0
	rs112339338	*33	Missense (R163C)	<0.1	<0.1	0.2	0	0	0	<0.1	<0.1	0
	rs72552739		Stop gain (E98X)	<0.1	<0.1	<0.1	0	0.2	0	0	0	0
	rs1446592306		Missense (F40L)	<0.1	0	0	0	0	0	0	0.1	0
	rs759836180	*42	Frameshift	<0.1	<0.1	0	0.1	0	0	0	0	0

*EUR* European, *AFR* African, *FIN* Finnish, *SWE* Swedish, *EA* East Asian, *SA* South Asian, *LAT* Latino, *AJ* Ashkenazi Jew, *TPMT* thiopurine S-methyltransferase, *DPYD* encoding DPD dihydropyrimidine dehydrogenase.

Deleterious variants with frequencies above 0.1% in at least one population are shown.

For *TPMT*, only the TPMT*3 sub-alleles *3A and *3C, comprising Y240C alone or in combination with A154T, were common in at least one population, whereas *3B (only A154T) appeared very rare in all populations studied (MAF < 0.1%). TPMT*3A is common among Europeans (MAF = 3.8%), Latinos (MAF = 4.3%) and Ashkenazi Jews (MAF = 1.3%), while TPMT*3C is most abundant in Africa (MAF = 4.8%). In total, 17 *DPYD* and *TPMT* variants that impact enzyme function had allele frequencies above 0.1% in at least one population studied that might warrant inclusion into pharmacogenomic test panels.

### Population-specific differences in DPD and TPMT activity profiles

**Fig. 3:**
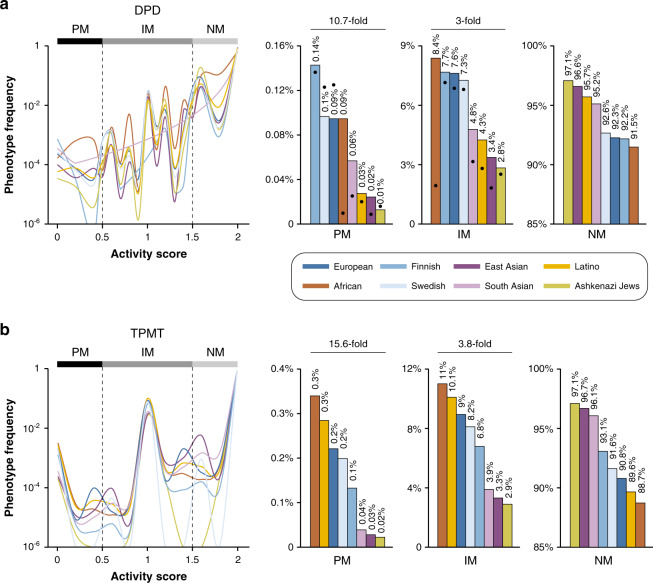
Integrative analysis of population-scale sequencing data reveals striking ethnogeographic differences in DPD and TPMT activity. Line plots (left) show population-specific aggregation of variant functionality scores into spectra of DPD (**a**) and TPMT (**b**) functionality. Column plots (right) indicate the fraction of poor metabolisers (PM), intermediate metabolisers (IM) and normal metabolisers (NM) for each population. Overlaid dots in panel **a** indicate predictions based on available variant activity scores of DPYD-Varifier (training and test sets). As this algorithm only provides binary assessments, all deleterious and neutral variants were considered to have functionality scores of 0 and 1, respectively.

By integrating the predicted quantitative allele activities of all identified *DPYD* and *TPMT* variations with their population-specific frequencies, we provide the first quantitative spectrum of functional variability across eight different populations. Our predictions showed that Finns harboured the highest number of DPD-poor metabolisers with frequencies of 0.14%, primarily due to high frequencies of DPYD*2A in this population (MAF = 2.4% compared to 0.6% in Europeans, Table 2), whereas Africans were most likely to have intermediate DPD activity with frequencies of 8.4% (Fig. 3a). While 0.09% and 7.6% of Europeans were classified as PMs and IMs, they are much lower among Ashkenazi Jews (0.01% PMs and 2.8% IMs), in agreement with previous reports showing considerably lower frequencies of D949V in Ashkenazim compared to Europeans.^42^ Interestingly, substantial differences in DPD metabolic phenotypes were found between Asian populations with the predicted incidence of PM phenotypes being threefold higher in South Asia compared to East Asia. Combined, these data reveal surprisingly large ethnogeographic differences in DPD phenotypic profiles with PM and IM incidence differing 10.7 and 3-fold between populations.

Next, we compared these results to predictions obtained using DPYD-Varifier, a recently developed machine-learning algorithm specifically trained for *DPYD* variant classification.^26^ Notably, population-specific frequencies of DPD metaboliser phenotypes were overall in good agreement. However, drastic differences were observed for Africans, for whom DPYD-Varifier underpredicted PM and IM frequencies by nine- and fourfold, respectively, compared to APF. To analyse the underlying reasons, we compared variant classifications and found that Y186C, an African-specific variant with a frequency of 2%, only showed minor reductions in DPYD-Varifier training data (85% activity of WT), whereas it was predicted to be deleterious by APF (20% activity). Importantly, Y186C is associated with decreased DPD activity and severe fluoropyrimidine toxicity in vivo.^43–45^

Compared to the frequency of reduced DPD activity phenotypes, the incidence of PM and IM TPMT metabolisers was considerably higher (Fig. 3b). Reduced TPMT activity was most common in Africans (PM = 0.3% and IM = 11%) and Latin Americans (PM = 0.3% and IM = 10.1%), whereas the incidence in Ashkenazim was multiple-fold lower (PM = 0.02% and IM = 2.9%). Different from DPD, no significant phenotype difference was found within European and Asian populations.

## Discussion

DPD and TPMT deficiency are the major determinants of severe fluoropyrimidine and thiopurine-associated toxicity, and prospective genotyping followed by genotype-guided prescribing has been shown to reduce adverse events.^12,46^ Thus, the identification of genetic variations that result in reduced enzyme function is of central importance to improve patient outcomes. A few dozen variants have well-characterised effects in vivo and can be acted on accordingly once they are identified in a patient’s genome. However, previous sequencing efforts, such as the 1000 Genomes Project (*n* = 2504 individuals) and the Exome Sequencing Project (*n* = 6503 individuals), identified >100 additional *DPYD* and *TPMT* variations with unclear functional consequences. Consequently, while rare genetic variations have long been considered important factors to explain at least part of the missing heritability in drug response,^47,48^ there are currently no guidelines for carriers of such uncharacterised variants as to whether or not to modify doses or switch to alternative agents. There is thus a need for computational tools that can aid in the reliable functional interpretation of such variations.

Our data showed that commonly used non-pharmacogene-specific algorithms that perform well on disease data^49^ had only moderate predictive power for *DPYD* and *TPMT* variants (62.1–78.6%). In contrast, DPYD-Varifier, a machine-learning-based classifier trained exclusively on *DPYD* variant data, showed 85% accuracy on a set of novel missense variations compared to in vitro data.^26^ Here, we find that APF, a quantitative ensemble score we recently developed specifically for pharmacogenetic predictions,^23^ achieved a binary classification accuracy of 91.4% on a set of 70 variations with known in vivo effects. However, we want to emphasise that these results cannot be directly compared to DPYD-Varifier, as all these variants in question were used for the training of this tool. In contrast, APF has not been trained on any *DPYD* variants, suggesting that the underlying framework is broadly applicable to the functional interpretation of variants across pharmacogenes encoding metabolic enzymes. Few discrepancies between the in vitro assessment and in vivo function were reported for some variants, such as Y186C and D974V. Consequently, we argue for the benchmarking of computational tools on variants with known effects in vivo.

While APF constitutes the only tool providing quantitative estimates of variant function across pharmacogenes, it also has notable limitations. APF cannot detect gain-of-function effects and variants that result in increased enzymatic function in vitro, such as DPD P1023T,^24^ which APF classifies as functionally neutral. In addition, APF is designed for the analysis of individual variants. As such, the functionality of variant combinations is driven by the most deleterious variant in the haplotype. However, a recent clinical study showed that a *DPYD* haplotype containing three neutral missense variants (C29R, M166V and V732I) is strongly associated with decreased reduced fluorouracil degradation and severe toxicity,^50^ which would thus be missed by APF. Furthermore, we note that APF results in an excess of false positives for *DPYD*, such as D829N, A450V and S534N, which are correctly classified as functionally neutral using in vitro assays. By contrast, APF correctly flagged *DPYD* Y186C and D974V as deleterious, whereas in vitro studies only detected minor reduction of 15 and 22% of WT DPD activity for these variants, respectively.^24^

While *DPYD* V732I (DPYD*6) is mostly considered as benign by both in vivo and in vitro studies,^7,51,52^ as well as our APF algorithm, this variant has recently been implicated in increased risk of gastrointestinal and haematological fluoropyrimidine toxicity.^53,54^ Importantly, unlike other algorithms, APF provides an activity score estimate that strongly correlates with measured in vitro activity (*R*^2^ = 0.9; *P* = 2.9 × 10^−5^).^23^ V732I was estimated to have an activity score of 0.6, slightly above the selected binary classification threshold for deleteriousness of 0.5, suggesting that also variants with modest decreases in activity might increase toxicity risks, albeit with lower risk scores than clear loss-of-function variants, such as DPYD*2A and D949V (APF score of 0 for both). This is consistent with the findings by Del Re et al.^54^ and Boige et al.^53^ who reported only moderate hazard ratios (HRs) for V732I, D949V of 1.7–1.9, respectively, whereas HRs for DPYD*2A were substantially higher (6.3 and 4.2, respectively).

Multiple challenges need to be overcome before fluoropyrimidine and thiopurine dosing based on personalised genomic profiles and their computational interpretations can be implemented in clinical practice. Most importantly, implementation efforts critically rely on the demonstration of clinical utility using stringent prospective trials. In addition, the establishment of population-scale genomic biobanks^55–57^ with associated longitudinal electronic medical records offers exciting opportunities to test the predictive power of this and other computational prediction algorithms retrospectively. However, even in the absence of such data, we believe that the current algorithms are already sufficiently accurate to flag patients with putatively deleterious but experimentally uncharacterised variations for increased monitoring. In addition to the clinical validity of in silico predictions, decisions of gene sequencing require careful evaluations regarding the cost-effectiveness of such measures compared to conventional genotyping, particularly as not even these candidate interrogations are routinely conducted in most countries.

By leveraging APF’s scalability, we provide the first population-scale profiles of DPD and TPMT metaboliser phenotypes that consider the entire repertoire of coding genetic variation. Importantly, our analyses considered all variants affecting the amino acid sequence of the respective gene product (missense, start-lost, frameshifts, splicing and stop gain), as well as the intronic variant rs75017182 (*DPYD* HapB3). Uracil breath tests and peripheral blood mononuclear cell DPD radioassays indicated a prevalence of DPD deficiency in Africans of 8%,^58^ which aligns very well with our APF estimates of 8.4%. Furthermore, our data suggested a prevalence of reduced DPD function alleles in Europeans of 7.6%, again in good agreement with the results of a prospective study in the Netherlands, which reported 8% of patients to carry at least one functionally relevant *DPYD* variant allele.^12^ A previous study indicated that DPD deficiency due to DPYD*2 *A* was very high in Sweden with MAF = 3.5%,^59^ while our analysis of 1000 Swedish genomes showed lower frequencies of 0.8%. Notably, however, *2A frequencies in neighbouring Finland (*n* = 12,562) were substantially higher (MAF = 2.4%), corroborating an overall high rate of DPD deficiency in Scandinavia. For TPMT, the available literature indicates PM and IM frequencies of 0.45–0.6% and 9.9–11.9% for Caucasians,^15,60–63^ again closely corresponding to APF predictions of 0.2% and 9%. Notably, while genotype-based predictions align overall well with measured TPMT phenotypes (97% concordance), concordance is lower for TPMT*1/*2 and *1/*3 heterozygotes (79% concordance).^64^

In summary, this study demonstrates that the pharmacogenetically trained APF classifier provides accurate predictions of *DPYD* and *TPMT* variant functionality outperforming conventionally used algorithms trained on disease data. We show that DPD metaboliser status is genetically complex and requires profiling of tens of variations to explain the majority of phenotypic differences. In contrast, >95% of functional TPMT variability is explained by only four variants. By leveraging population-scale sequencing data, we provide spectra of ethnogeographic variation in DPD and TPMT phenotypes on an unprecedented scale, and reveal unexpectedly large interethnic differences in DPD and TPMT deficiencies. Our results provide a powerful resource for the worldwide distribution of the major genetic determinants of fluoropyrimidine and thiopurine metabolism with important implications for population-adjusted genotyping strategies and precision public health.

## Supplementary information

Supplementary table 1 - REVISED

## Data Availability

All *DPYD* and *TPMT* variants with available in vivo data analysed in this study are provided in Supplementary Table S1. Sequencing data of 138,842 individuals are available at https://gnomad.broadinstitute.org/.
